# Improved Recognition of Heart Attack and Stroke Symptoms After a Community-Based Intervention for Older Adults, Georgia, 2006-2007

**Published:** 2009-03-15

**Authors:** Mary Ann Johnson, Mindy Bell, Tiffany Lommel, Joan G. Fischer, Jung Sun Lee, Sudha Reddy

**Affiliations:** University of Georgia, Department of Foods and Nutrition; University of Georgia, Athens, Georgia; University of Georgia, Athens, Georgia; University of Georgia, Athens, Georgia; University of Georgia, Athens, Georgia; Georgia Department of Human Resources, Atlanta, Georgia

## Abstract

**Introduction:**

In Georgia, mortality from stroke is 16% higher and from cardiovascular disease is 9% higher than it is nationally. Although 75% of Georgia adults have 2 or more modifiable risk factors for cardiovascular disease, less than half recognize all major heart attack and stroke warning symptoms. To reduce disability and prevent death from cardiovascular events, high-risk population groups should be able to recognize symptoms and seek immediate medical attention.

**Methods:**

We evaluated a 4-month education intervention in 40 senior centers in Georgia. The intervention focused on improving knowledge of heart attack and stroke symptoms and on promoting lifestyle behaviors that prevent and manage cardiovascular disease and diabetes. Participants in a convenience sample completed a pretest questionnaire, the intervention, and a posttest questionnaire (N = 693, mean age, 75 years, 84% female, 45% black).

**Results:**

After the intervention, recognition of all 5 symptoms of heart attack increased from 29% at the pretest to 46% at the posttest, and recognition of all 5 symptoms of stroke increased from 42% at the pretest to 65% at the posttest (for both conditions, *P* < .001). In linear regression analyses, independent positive predictors of change in knowledge were younger age and higher education. Most risk factors for cardiovascular disease were not predictive.

**Conclusion:**

The results of this evaluation provide an evidence base for the effectiveness of this intervention in improving knowledge about heart attack and stroke symptoms, which may translate to greater preparedness in these older adults for response to cardiovascular events.

## Introduction

The cardiovascular disease (CVD) death rate in Georgia is 9% higher than the national rate, and Georgia is within the geographic "stroke belt," the southeastern region of the United States defined by high stroke mortality compared with other US regions (1-3). Diabetes is a risk factor for CVD (1-4). The risk of stroke more than doubles each decade after age 55, and the risk is increased by health disparities (1-3). The prevalence of contributors to health disparities, such as low socioeconomic status, low education, living in a rural area, and belonging to a racial/ethnic minority group, is high in older Georgians (5), particularly among participants in Georgia's Older Americans Act (OAA) programs (6).

OAA programs provide supportive home- and community-based services, including helping older people stay active and healthy by preventing disease and disability through evidence-based programs (7-9). The OAA network includes 56 State Units on Aging, 655 Area Agencies on Aging (AAA), 236 tribal and native organizations, and thousands of senior centers, adult day care centers, service providers, caregivers, and volunteers (7-9). OAA programs are targeted to people who are aged 60 years or older and who have the greatest social and economic need. Compared with national averages, OAA participants are nearly 3 times more likely to meet federal guidelines for poverty, and OAA participants are more than twice as likely to be in poverty if they belong to a minority group (52%) compared with whites (21%) (8). Compared with national OAA participants, Georgia's participants are more likely to live in poverty (27.0% vs 46.2%) and to belong to a minority group (19.8% vs 36.8%) (6). Thus, a vulnerable population of older adults at high risk for CVD and stroke is served by OAA's integrated delivery of health promotion services.

Recognition of symptoms is one of the first steps to getting immediate medical care, which can reduce disability and prevent death. Knowledge of heart attack and stroke symptoms is lacking among Americans and is lowest among those at highest risk for CVD, including older people and racial minorities (10-12). Although 75% of adult Georgians have 2 or more modifiable CVD risk factors, less than half of all Georgians recognize the major symptoms of heart attack and stroke (13).

Georgia's OAA Nutrition Program (OAANP) has developed a statewide network of wellness coordinators in each of its 12 AAAs. This network has implemented and evaluated community-based nutrition and wellness programs that led to improved diabetes self-management, increased fruit and vegetable consumption, and increased physical activity and physical function (14-16). To address the urgent need to improve knowledge and behaviors related to CVD risk factors, a diabetes and heart health education intervention was developed, implemented, and evaluated in OAANP in senior centers across Georgia. This theory-based intervention focused on improving knowledge of risk factors and symptoms for heart attack and stroke. We report the findings of this program evaluation.

## Methods

### Sample

Procedures were approved by the institutional review board on human subjects of the Georgia Department of Human Resources and the University of Georgia (UGA). Wellness coordinators, senior center directors, and their staff recruited a convenience sample of older adults enrolled in the OAANP from 40 senior centers in Georgia. Most participants were recipients of congregate meals, which are meals that are provided at the senior centers and paid for primarily from local, state, and/or federal funds as part of the services offered at the center. Potential participants were excluded if they were homebound or demonstrating cognitive impairment as determined by the interviewer's assessment. Approximately 3,500 people participated in some aspect of this intervention, of whom 849 were formally enrolled and participated in the pretest evaluation (approximately 70 participants per AAA). Consent forms were read to participants and written informed consent was obtained.

### Pretest

In collaboration with the Division of Aging Services and the wellness coordinators, 3 faculty and staff from the UGA Department of Foods and Nutrition developed and edited the pretest and posttest questionnaires to ensure content validity and cultural appropriateness based on their collective experience in working with the target population. Pretests were administered in November and December 2006. The study was explained, informed consent was obtained, and pretests were completed in approximately 1 hour. Trained wellness coordinators and other staff affiliated with the senior centers read the questions to participants and recorded their responses. Demographic characteristics, general health (eg, self-reported diabetes, hypertension, heart disease, high cholesterol) (17), and height and weight (measured or self-reported) were assessed. Body mass index (BMI) was reported as weight in kilograms divided by height in meters squared. Knowledge of 6 heart attack and 6 stroke symptoms, and actions to take should these events occur (eg, calling 9-1-1), was assessed with questions from the Behavioral Risk Factor Surveillance System (BRFSS) questionnaire (17). A sample question is, "Do you think pain or discomfort in the jaw, neck, or back are symptoms of a heart attack?" (Yes/no/don't know). Each set of 6 questions on heart attack and stroke symptoms included 1 incorrect, or "decoy" symptom to assess participants' ability to discriminate among true and false symptoms. Summary scores of total symptoms correctly identified were computed as mean number of questions answered correctly (range 0 to 6). For both the heart attack and stroke questionnaires, responses of "don't know" were combined with incorrect responses, and summary scores were computed for participants who provided complete data for all symptoms for each condition. The effect of the intervention on other variables, such as physical activity and diet, is summarized elsewhere (M. A. Bell, MS, written communication, August 2008).

### Intervention

After participants completed pretest questionnaires, they participated in the educational intervention, which consisted of 8 lessons about diabetes and heart health given during 16 weeks (January through April 2007). Each lesson was given once, lasted 45 to 60 minutes, and incorporated physical activity. With input from the Georgia Division of Aging Services, the intervention curriculum was developed by faculty and staff in the UGA Department of Foods and Nutrition who have experience with the target population and who ensured that the curriculum was accurate, culturally appropriate, safe for participants, and could be delivered by people who were well educated but who were not necessarily health professionals. The curriculum was developed on the basis of our previous experience with interventions designed to improve physical activity, fruit and vegetable intake, and diabetes self-management (14-16) and can be accessed online (18).

The conceptual framework for this intervention was based on the health belief model (19). The intervention incorporated key components of this framework, including perceived susceptibility and severity (eg, CVD and diabetes risk factors and related complications that frequently affect older adults and are associated with lifestyle habits), perceived benefits (eg, improved outcomes for getting immediate treatment for heart attack or stroke), perceived barriers (eg, information and misinformation about CVD and diabetes, heart attack and stroke symptoms, and lifestyle behaviors, such as diet and physical activity), cues to action, and self-efficacy.

Participants attended up to 8 sessions on diabetes and heart health and up to 8 sessions on bone health, discussed elsewhere (J. Teems, MS, written communication, August 2008). The 2 interventions were given on alternate weeks, and all 16 sessions promoted physical activity. The diabetes and heart health curriculum highlighted 8 key messages that were introduced at the first lesson (eg, "Know Warning Signs for Heart Attack, Stroke, and Diabetes") and reiterated at the next 7 lessons (Table 1).

### Posttest

The posttest was administered 1 to 2 months after the last lesson (May and June 2007). The posttest was similar to the pretest, except that questions were added to allow participants to further describe behavior changes and to rate the program.

### Statistical analyses

Pretest and posttest questionnaires were sent by the wellness coordinators to UGA for data entry, and SAS version 9.1 (SAS Institute, Inc, Cary, North Carolina) was used for analysis. Surveys that were missing responses for any of the 6 individual questions at the pretest or posttest were not assigned a summary score for heart attack (n = 42) or stroke (n = 54). Data from the pretest and posttest were compared by using paired *t* tests and χ^2^ analyses. Regression analyses were used to explore the predictors of pretest knowledge and changes in knowledge for symptoms of heart attack and stroke (6-item summary scores). Variables included in these models were pretest demographics, self-reported health, and self-reported CVD risk factors (overweight, obesity, diabetes, tobacco use, heart disease, high blood pressure, and high cholesterol), as well as the summary score for heart attack and stroke knowledge at the pretest for the models that explored changes in knowledge after the intervention. Differences were considered significant at *P* ≤ .05.

## Results

Of the 849 participants who completed pretest questionnaires, 82% completed the posttest questionnaires. Participants did not complete the posttest for the following reasons: cognitive impairment (n = 2), homebound (n = 7), deceased (n = 8), refused (n = 19), hospitalized/sick (n = 26), no reason given (n = 37), or no longer attended the senior center (n = 57). Thus, the final sample size for statistical analyses of the posttest changes was 693. Some of the analyses have fewer than 693 participants because of incomplete responses on other variables. Participants who did not complete the posttest (n = 156) were not significantly different than those who did (n = 693) except for their age; those who did not complete the posttest were significantly younger than those who did (mean age, 73 years vs 75 years, *P* = .02).

Mean age of the study participants who completed both pretest and posttest was 75 years, most participants were female, almost half were African American, most had a BMI in the overweight or obese categories (≥25.0 kg/m^2^) (20), and the prevalence of self-reported health conditions was high (Table 2). People with diabetes were intentionally oversampled, with a goal of at least 20 of 70 people in each AAA.

Correct identification of the symptoms of a heart attack ranged from 30% to 87% at the pretest and from 35% to 92% at the posttest, and all individual measures and summary scores significantly improved after the intervention (Table 3). Of the 6 symptoms, the mean (standard deviation) correctly identified was 3.7 (1.5) at the pretest and 4.3 (1.4) at the posttest. Pretest knowledge of individual symptoms was comparable to the patterns of 2001 BRFSS data in similar age groups (10-12). After the intervention, 46% of participants recognized all 5 symptoms of a heart attack, an increase of 17 percentage points.

Correct identification of the symptoms of stroke ranged from 18% to 87% at the pretest and from 34% to 94% at the posttest, and all individual measures and mean summary scores significantly improved after the intervention (Table 4). Of the 6 symptoms, the mean (SD) correctly identified was 3.9 (1.6) at the pretest and 4.6 (1.4) at the posttest. Pretest knowledge of the individual symptoms was comparable to the patterns of 2001 BRFSS data in similar age groups (11). After the intervention, 65% of participants recognized all 5 symptoms of a stroke, an increase of 27 percentage points.

Linear regression models were developed to explore demographic and health-related predictors of the 6-item knowledge scores for heart attack symptoms and for stroke symptoms at the pretest and for changes from the pretest to the posttest. Only participants who had complete data for all selected potential predictors were included in these analyses (demographics, self-reported health, BMI, and self-reported diabetes, heart disease, high blood pressure, and high cholesterol, n = 542). Higher knowledge score for heart attack at pretest was significantly associated with being younger (*P* = .04) and with being white (vs black, *P* < .001). Higher knowledge score for stroke at pretest was associated with being younger (*P* < .01), being white (vs black, *P* < .001), and higher education (*P* < .001; data not shown).

In the regression models, when controlled for knowledge at the pretest, improvements in heart attack knowledge were significantly associated with being young (*P* < .001), being highly educated (*P* < .01), and reporting high blood pressure (*P* = .04). Improvements in stroke knowledge were significantly associated with being young (*P* < .001), being white (vs black, *P* < .01), and being highly educated (*P* < .001). Similar results were found in regression analyses using the 5-item summary scores for heart attack and stroke symptoms (without the "decoy" question; data not shown).

Participants attended approximately 75% of the sessions. Overall satisfaction with the education and physical activity programs was high among those who completed the posttest; 32% rated the education program as excellent, 40% as very good, 24% as good, 4% as fair, and <1% as poor. Eighty-nine percent of respondents indicated that they had learned the warning signs of a heart attack and of a stroke during the intervention.

## Discussion

In this community-based intervention, young age, white race, and high education level were associated with better knowledge of heart attack and stroke symptoms at pretest and with changes in knowledge after the intervention. However, the presence of risk factors and behaviors related to heart attack and stroke, such as heart disease, diabetes, and tobacco use, were not consistently associated with knowledge at pretest or improvements in knowledge. Thus, given the overall increases in knowledge, this 4-month diabetes and heart health intervention was well-targeted to a vulnerable population of older adults at high risk for CVD. This population can be readily accessed through the aging network, one of whose goals is to help older people stay active and healthy through evidence-based disease and disability prevention programs (7-9). The intervention is consistent with *Healthy People 2010* objectives of increasing awareness of the early warning signs and symptoms of heart attack and stroke and accessing rapid emergency care by calling 9-1-1 (21).

Knowledge of heart attack and stroke symptoms was assessed with prompting questions from the BRFSS, which may have overestimated knowledge compared with open-ended questions that require recall of symptoms (22-23). Overall, pretest knowledge of heart attack in our study was comparable to the patterns seen with older participants in 17 states in the 2001 BRFSS and in 14 states in the 2005 BRFSS (10,12). Only a small percentage of participants in our intervention knew all symptoms of a heart attack, including the incorrect symptom, and also knew that calling 9-1-1 should be the first action in response to a possible heart attack. Similar to our study, others report that being a racial/ethnic minority, being a man, having low socioeconomic status, and having low education is predictive of low knowledge of heart attack symptoms; however, having risk factors for heart attack is generally not related to knowledge (10,12,22). We did not find a relationship between sex and knowledge, perhaps because of the small number of men in our sample (16% men, Table 2).

Pretest stroke knowledge also was comparable to that seen in the 2001 BRFSS data, which demonstrated a range of knowledge across individual symptoms (11). In our sample, people who were old, black, and who were not well educated had a low awareness of symptoms (*P* < .05, regression analyses). Our findings raise concern because people with risk factors for CVD, who are often the majority of older people and minority groups, are the most vulnerable to heart attack and stroke. In Georgia, the age-adjusted death rate for stroke is 1.4 times higher among blacks than whites, and people aged 65 and older account for 81% of stroke deaths (2). Many senior centers were located in rural areas (2,5).

Although community interventions and public health campaigns help improve knowledge of heart attack and stroke (24,25), the decision to seek immediate medical care is often complex and multifactorial (11,12,26-30). Prehospital delay and help-seeking behavior were outcomes beyond the scope of our study. However, many clinical, social, emotional, cognitive, demographic, and other factors related to and outside of knowledge may influence delay time, such as presence of chronic conditions, embarrassment about seeking help, calling one's physician, slow onset of symptoms, and failure to recognize atypical symptoms (27-30). Older age, low socioeconomic status, and female sex have also been associated with increased delay time (27-31). Therefore, future studies should address barriers to immediate action and factors that may hinder the application of knowledge among high-risk groups (27).

This study has several limitations. Participants were part of a convenience sample with no parallel control group. Randomized, controlled studies are needed. Differences among the educators in backgrounds (eg, dietitians, nurses, and recreational therapists), styles of delivery, and adherence to protocol across the state could have led to variation in how the intervention was delivered. However, we did use several quality control measures, including a statewide training session and technical assistance on site and by phone and e-mail. Lesson plans were scripted in an easily understandable and culturally relevant manner on the basis of previous experience with the target population, and educators were encouraged to be sensitive to the learning abilities of their audience and to emphasize the materials that best met the needs of their participants. This study relied primarily on self-report measures, including most of the height measures and about 23% of the weight measures. The posttests were completed within 1 to 2 months after the intervention. Future studies might include posttests conducted at later dates following the intervention and continued reinforcement of symptom knowledge on a regular basis. An unexpected increase in incorrectly answering the decoy question for signs and symptoms of heart attack was seen at posttest. Other researchers have noted that the format in which such questions are asked can influence participants' responses; the format we used tends to yield a higher number of correct responses compared with using a recall-only method without prompting (23). Future interventions should place more attention on the desired responses, such as calling 9-1-1.

This intervention can be replicated using our educational materials, which are available online free of charge (18). Costs associated with this intervention were not specifically calculated but were estimated to be approximately $200,000 (funded by the state's "Live Healthy Georgia" campaign) for educational materials, training interviewers and educators, technical site visits, personnel costs, and data entry and statistical analyses. Many additional costs were absorbed by the UGA Department of Foods and Nutrition (eg, gratis graduate student and faculty time for developing the educational materials, and writing and submitting manuscripts), volunteers, and the aging network that is well established in Georgia, where more than 200 senior centers and other providers offer more than 34,000 seniors annually with nutrition and wellness services (32).

Continued reinforcement of these educational messages is needed. We observed a significant increase after the intervention in the percentage of participants who recognized all individual symptoms of heart attack or of a stroke, but perhaps modification of the program is needed to improve recognition above that observed for identification of the 5 true signs and symptoms (46% and 65% for heart attack and stroke, respectively, at posttest). To help reinforce and personalize public health messages delivered at the community level, health professionals should routinely educate high-risk patients and their caregivers about symptoms of heart attack and stroke, the benefits of prompt medical attention, and strategies to overcome barriers to seeking immediate care (26-31). Future efforts may include 1) reinforcement of heart health messages, 2) greater community involvement in the delivery of the intervention by inviting emergency medical services (EMS) staff to address concerns participants may have about accessing EMS and seeking medical attention, and 3) linking this intervention to other state initiatives, such as the Cardiovascular Health Initiative and the Stroke and Heart Attack Prevention Program (1).

## Figures and Tables

**Table 1:** Diabetes and Heart Health Intervention Curriculum, Georgia Senior Centers, 2006-2007^a^

Lesson No. and Title	Topics
1. My Eight Ways to Feel Great	Definitions of CVD and diabetes and their prevalence Eight goals to prevent and manage these conditions; 1 of the goals was knowing the major warnings signs of heart attack, stroke, and diabetes
2. Be Physically Active Every Day	The health benefits of regular physical activity for people with diabetes and CVD Strategies to incorporate physical activity into a healthy lifestyle
3. Healthy Eating — Up With Fruits, Vegetables, and Whole Grains, Down with Fat and Sodium	Ways to include a variety of healthy plant foods in daily meals and snacks How to limit intake of nutrients that can increase the risk of CVD, such as saturated fat and sodium
4. Healthy Eating — Control Portions and Choose a Variety of Foods	The "plate method" to plan balanced meals using sensible portions of nutritious foods How to read food labels to choose foods with less saturated fat, added salt, and sugar
5. Prevent and Manage Heart Disease, Stroke, and Diabetes	Ways to prevent and manage CVD and diabetes by controlling major risk factors The major warning signs of heart attack, stroke, and diabetes
6. Get Checked for Diabetes and Heart Disease Risk Factors	The importance of recommended health screenings to control risk factors and complications related to CVD and diabetes
7. Managing My Medications	How to manage medications by talking to a doctor and pharmacist How to increase safety and organization for taking medications as recommended
8. Know the Warning Signs of Heart Attack, Stroke, and Diabetes	Detailed discussion of warning signs of heart attack, stroke, and diabetes, and emergency procedures to follow should these events occur Information available from the National Heart Lung and Blood Institute and the National Institute for Neurological Disorders and Stroke

Abbreviation: CVD, cardiovascular disease.

a The complete curriculum is titled "Seniors Taking Charge of Diabetes and Heart Health" and is available online free of charge at http://www.livewellagewell.info/study/materials.htm#2007.

**Table 2:** Characteristics of Participants Who Completed Pretest and Posttest Questionnaires, Diabetes and Heart Health Intervention, Georgia Senior Centers, 2006-2007 (N = 693)

**Characteristic**	Mean (SD) or %^a^
**Age, y **	75 (7.8)
≤69	27
70-79	46
≥80	27
**Sex **	
Male	16
Female	84
**Race/ethnicity **	
White	54
Black	45
Other	<1
**Education, y (n = 692)**	10.5 (3.2)
**Body mass index, kg/m^2^ (n = 678)**	29.6 (6.5)
<25.0	25
25.0 to 29.9 (overweight)	35
≥30.0 (obese)	41
**Waist circumference, in (n = 675)**	
Men	40.5 (5.2)
Women	38.8 (5.7)
**Tobacco use (n = 681)**	9
**Self-reported health conditions^b^**	
Diabetes^c^ (n = 688)	36
High blood pressure (n = 685)	73
Heart disease (n = 685)	31
High cholesterol (n = 692)	55
Arthritis (n = 690)	73
**Self-reported health (n = 691)**	
Poor	6
Fair	32
Good	43
Very good	15
Excellent	4

Abbreviation: SD, standard deviation.

a Percentages may not add to 100 because of rounding.

b Values add to more than 100 because some respondents reported more than 1 health condition.

c People with diabetes were oversampled, so value may represent an overestimate of the true prevalence.

**Table 3:** Changes in Knowledge of Heart Attack Symptoms After the Diabetes and Heart Health Intervention, Georgia Senior Centers, 2006-2007 (N = 693)

**Heart Attack Symptom/Survey Response Item^a^**	Pretest Mean^b^	Posttest Mean^b^	Change Mean^b^,^c^	*P* Value^d^
**Pain or discomfort in the jaw, neck, or back (n = 682)**				
Yes^e^	49	66	17	<.001
No	18	14	−4	
Don't know	33	20	−13	
**Feeling weak, lightheaded, or faint (n = 677)**				
Yes^e^	51	65	14	<.001
No	20	16	−4	
Don't know	30	18	−12	
**Chest pain or discomfort (n = 675)**				
Yes^e^	87	92	5	.02
No	3	3	0	
Don't know	9	5	−4	
**Sudden trouble seeing in 1 or both eyes (n = 676)**				
Yes	24	36	12	<.001
No^e^	30	35	5	
Don't know	46	29	−17	
**Pain or discomfort in the arms or shoulder (n = 677)**				
Yes^e^	76	86	10	<.001
No	8	6	−2	
Don't know	16	8	−8	
**Shortness of breath (n = 681)**				
Yes^e^	78	86	8	<.001
No	6	6	0	
Don't know	16	9	−7	
**Mean no. of correct answers^f^ (SD) (n = 651)**	3.7 (1.5)	4.3 (1.4)	0.6 (1.6)	<.001
**Frequency of correct answers (n = 651)**				
<5	62	42	−20	<.001
≥5	38	58	20	
All 6 correct (n = 651)	7	14	7	<.001
All 5 true signs and symptoms correct (excluding decoy symptom) (n = 651)	29	46	17	<.001
**Know to call 9-1-1 if someone was having a heart attack or stroke (n = 680)**	84	92	8	<.001

Abbreviation: SD, standard deviation.

a Possible answers to the question, "Which of the following do you think is a symptom of a heart attack?"

b All values are percentages unless otherwise stated; percentages may not add up to 100 because of rounding.

c Completed both the pretest and posttest; differences may not equal posttest minus pretest because of rounding.

d *P* values calculated with paired *t* tests for differences in means and χ^2^ analyses for differences in percentages between the pretest and posttest.

e Correct response to question.

f A response of "don't know" was included with incorrect responses. The maximum score was 6.

**Table 4:** Changes in Knowledge of Stroke Symptoms After the Diabetes and Heart Health Intervention, Georgia Senior Centers, 2006-2007 (N = 693)

Stroke Symptom/Survey Response Item^a^	Pretest Mean^b^	Posttest Mean^b^	Change Mean^b^,^c^	*P* Value^d^
**Sudden confusion or trouble speaking (n = 679)**				
Yes^e^	86	92	6	.002
No	3	3	0	
Don't know	11	5	−6	
**Sudden numbness or weakness of face, arm, or leg, especially on 1 side (n = 678)**				
Yes^e^	87	94	7	<.001
No	2	2	0	
Don't know	11	5	−6	
**Sudden trouble seeing in 1 or both eyes (n = 669)**				
Yes^e^	58	75	17	<.001
No	9	7	−2	
Don't know	33	17	−16	
**Sudden chest pain or discomfort (n = 661)**				
Yes	46	43	−3	<.001
No^e^	18	34	16	
Don't know	36	23	−13	
**Sudden trouble walking, dizziness, or loss of balance (n = 681)**				
Yes^e^	74	85	11	<.001
No	6	5	−1	
Don't know	20	10	−10	
**Sudden severe headache with no known cause (n = 681)**				
Yes^e^	61	79	18	<.001
No	11	6	−5	
Don't know	28	14	−14	
**Mean no. of correct answers^f^ (n = 639)**	3.9 (1.6)	4.6 (1.4)	0.8 (1.7)	<.001
**Frequency of correct answers (n = 639)**				
<5	54	27	−27	<.001
≥5	46	73	27	
All 6 correct (n = 639)	8	22	14	<.001
All 5 true symptoms correct (excluding decoy symptom) (n = 639)	42	65	23	<.001
**Know to call 9-1-1 if someone was having a heart attack or stroke (n = 680)**	84	92	8	<.001

a Possible answers to the question, "Which of the following do you think is a symptom of a stroke?"

b All values are percentages unless otherwise stated; percentages may not add to 100 because of rounding.

c Completed both the pretest and posttest; differences may not equal posttest minus pretest because of rounding.

d *P* values calculated using paired *t* tests for differences in means and χ^2^ analyses for differences in percentages between the pretest and posttest.

e Correct response to question.

f A response of "don't know" was included with incorrect responses. The maximum score was 6.
